# Prostatic artery embolization for giant prostatic hyperplasia: a single-center experience

**DOI:** 10.1590/0100-3984.2020.0096

**Published:** 2021

**Authors:** André Moreira de Assis, Airton Mota Moreira, Francisco Cesar Carnevale, José Ramón Lanz-Luces

**Affiliations:** 1 Interventional Radiology Department, Instituto de Radiologia do Hospital das Clínicas da Faculdade de Medicina da Universidade de São Paulo (InRad/HC-FMUSP), São Paulo, SP, Brazil.

**Keywords:** Prostate/blood supply, Embolization, therapeutic, Prostatic hyperplasia/diagnostic imaging, Lower urinary tract symptoms, Severity of illness index, Quality of life, Próstata/irrigação sanguínea, Embolização terapêutica, Hiperplasia prostática/diagnóstico por imagem, Sintomas do trato urinário inferior, Índice de gravidade de doença, Qualidade de vida

## Abstract

**Objective:**

To describe the safety and efficacy of prostatic artery embolization (PAE) in patients with a markedly enlarged prostate.

**Materials and Methods:**

This was a retrospective study including 18 consecutive patients (mean age, 74 years) with benign prostatic hyperplasia, all with a prostate volume ≥ 200 cm^3^, who were enrolled to receive PAE for the treatment of moderate-to-severe lower urinary tract symptoms.

**Results:**

The PAE procedure was technically successful in 17 patients (94.4%). During follow-up, clinical failure (defined as an International Prostate Symptom Score [IPSS] ≥ 8) was observed in two (11.1%) of those 18 patients. At 3 months of follow-up, there was significant improvement over baseline in all relevant outcome measures: total IPSS (from 15.7 to 2.9); IPSS quality of life score (from 5.2 to 1.0); prostate specific antigen (from 11.4 to 1.82 ng/mL); peak urinary flow rate (from 7.45 to 18.6 mL/s); prostate volume (from 252.4 to 151.6 cm^3^); and post-void residual volume (from 143.7 to 28.3 mL)-*p* < 0.05 for all. Of the 18 patients, one (5.6%) presented detachment of prostate tissue and self-limited hematuria, which did not require specific treatment.

**Conclusion:**

In patients with a markedly enlarged prostate, PAE proved to be safe and effective, resulting in significant improvements in clinical, imaging, and urodynamic parameters.

## Abstract (pt)

**Objetivo:**

Descrever a segurança e eficácia da embolização das artérias prostáticas (EAP) em pacientes com próstatas muito aumentadas (≥ 200 cm^3^).

**Materiais e Métodos:**

Este estudo retrospectivo incluiu 18 pacientes consecutivos com hiperplasia prostática benigna portadores de próstatas ≥ 200 cm^3^ (idade média de 74 anos), que foram submetidos a EAP para tratar sintomas de trato urinário inferior moderados a graves.

**Resultados:**

A EAP foi tecnicamente bem-sucedida em 17 pacientes (94,4%). Falha clínica (IPSS ≥ 8) foi detectada em dois pacientes durante o seguimento (11,1%). Observamos melhora significativa em todos os parâmetros relevantes aos três meses de acompanhamento: IPSS: 15,7 vs. 2,9; qualidade de vida: 5,2 vs. 1,0); PSA: 11,4 vs. 1,82 ng/mL; pico de fluxo urinário: 7,45 vs. 18,6 mL/s); volume prostático: 252,4 vs. 151,6 cm^3^; e volume urinário residual: 143,7 vs. 28,3 mL - *p* < 0,05 para todos). Um paciente (5,6%) apresentou eliminação de tecido prostático e hematúria autolimitada durante o seguimento, que não necessitou de tratamento específico.

**Conclusão:**

A EAP em pacientes com próstata muito aumentada foi segura e eficaz, com significativas melhoras clínica, urodinâmica e imaginológica.

## INTRODUCTION

As the average age of the population continues to rise, mainly because of better living conditions-including improved health care, healthier nutrition, and early disease detection-physicians are facing aging-related conditions, including benign prostatic hyperplasia (BPH), with ever-increasing frequency. An enlarged prostate is common in men over 50 years of age and may or may not be accompanied by deleterious lower urinary tract symptoms (LUTS). In the absence of clinical signs, BPH can go undetected for quite some time, and the volume of the prostate can therefore increase significantly before appropriate treatment is given. In addition to age, risk factors for BPH include obesity, a condition that is prevalent worldwide, which is linked to metabolic syndrome and hormone imbalance, including changes in the ratio between circulating androgens and circulating estrogens^(1,2)^. Consequently, the aromatase enzyme that mediates the production of estrogens from testosterone^(3)^ could explain why testosterone levels in men drop by about 35% between the ages of 21 and 85, while estradiol levels either remain constant or increase. In rare cases, BPH can lead to a pronounced increase in prostate volume, resulting in a condition known as giant prostatic hyperplasia^(4,5)^.

Although surgical techniques such as holmium laser enucleation of the prostate and simple (open, robotic, or laparoscopic) prostatectomy have gained ground in dealing with severe prostate enlargement, they are associated with relevant morbidity, including retrograde ejaculation, intraoperative bleeding, urinary incontinence, and erectile dysfunction^(6)^, making it desirable to investigate minimally-invasive, low-morbidity alternative procedures. The objective of this study was to explore the clinical benefits, efficacy, and safety of prostatic artery embolization (PAE) in patients with a markedly enlarged prostate due to BPH.

## MATERIALS AND METHODS

**Figure 1:**
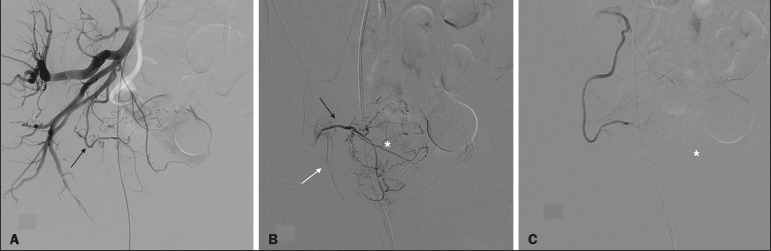
**A:** Digital subtraction angiography showing the right prostatic artery (arrow) originating from a common trunk with the superior vesical artery (type I origin). **B:** Superselective digital subtraction angiography of the right prostatic artery, showing the anteromedial branch (black arrow), the posterolateral branch (white arrow), and the hypervascularized right transitional zone (asterisk). **C:** Superselective digital subtraction angiography, performed after embolization, showing devascularization with no residual blush.

This was a retrospective study of 18 consecutive patients with BPH and a prostate volume ≥ 200 cm^3^, enrolled to receive PAE between March 2013 and May 2019 (Figure 1). Urologists referred all patients after considering PAE as an option for the treatment of LUTS. All participating patients gave written informed consent.

The medical staff assessed symptom severity and quality of life (QoL) by calculating the total International Prostate Symptom Score (IPSS) and the score on the IPSS QoL item, respectively^(7)^. The inclusion criteria were as follows: being ≥ 40 years of age; having a prostate volume ≥ 200 cm^3^; having been diagnosed with BPH; and presenting with a ≥ 6-month history of moderate-to-severe symptoms that were refractory to medical treatment, defined as an IPSS ≥ 8, or treatment-refractory acute urinary retention. Patients with histologically confirmed malignancy were excluded, as were those with large bladder diverticula, those with large bladder stones, those with chronic kidney disease, those with active urinary tract infection, and those with dysregulated coagulation.

The outcome measures, which were determined at baseline and at 3 months after PAE, were as follows: the total IPSS; the IPSS QoL item score; the prostate specific antigen (PSA) level; the peak urinary flow rate; prostate volume; and post-void residual volume (PVR), as determined by ultrasound. All patients also underwent magnetic resonance imaging (MRI) before PAE. The baseline characteristics of the patients are shown in Table 1.

**Table 1:** Baseline characteristics of patients with BPH.

Characteristic	Mean ± SD	Range
Age (years)	73.4 ± 9.0	60-92
IPSS	15.73 ± 4.2	9-22
IPSS QoL item score	5.16 ± 1.1	3-6
PSA (ng/mL)	11.97 ± 9.0	0.87-31
Peak urinary flow rate (mL/s)	7.49 ± 4.1	2-18
Prostate volume (cm^3^)	252.35 ± 44.9	202-348
PVR (mL)	143.72 ± 159.4	7-600

Technical success of the PAE was defined as bilateral embolization. Clinical failure was defined as an IPSS ≥ 8 or an IPSS QoL item score ≥ 3 at 3 months after the procedure, or inability to remove the indwelling catheter in patients with urinary retention. Complications were categorized in accordance with a modified version of the Clavien classification system for interventional radiology embolization procedures^(8)^.

### Imaging

**Figure 2:**
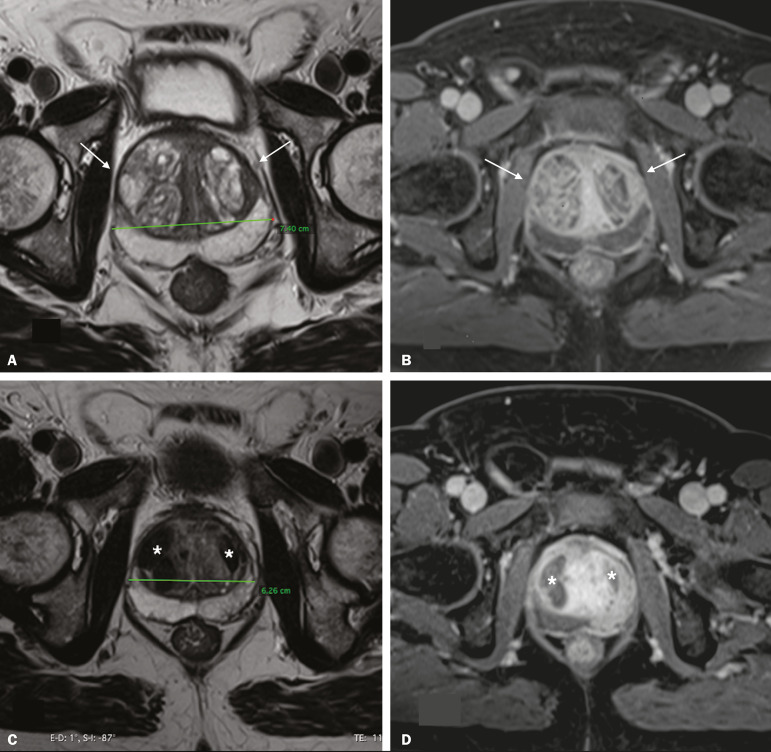
T2-weighted MRI sequence and contrast-enhanced T1-weighted MRI sequence with fat suppression (**A** and **B**, respectively), showing a markedly enlarged prostate due to bilateral BPH nodules in the transitional zone (arrows). **C,D:** T2-weighted MRI sequence and contrast-enhanced T1-weighted MRI sequence with fat suppression, both obtained after PAE (**C** and **D**, respectively), showing a reduction in prostate volume and infarction of the BPH nodules bilaterally (asterisks).

Before and after PAE, MRI of the prostate was performed in either a 1.5-T scanner or a 3.0-T scanner, with a pelvic phased-array coil (Figure 2). An endorectal coil was not used. Gadolinium-based contrast medium was administered with a power injector at a dose of 0.1 mL/kg or 0.2 mL/kg, followed by a 20-mL saline flush. Prostate volume was calculated by the prolate ellipsoid formula:

$$\mathrm{Prostate}\;\mathrm{volume}=\mathrm{length}\;x\;\mathrm{heigth}\;x\;\mathrm{width}\;x\;\left(\pi /6\right)$$

Two reviewers analyzed each MRI study, and disagreements were resolved by consensus, as recommended in the European Society of Urogenital Radiology guidelines^(9)^.

### PAE procedure

All patients underwent bilateral PAE according to previously described techniques^(10-12)^, the aim being the embolization of every single blood vessel feeding the prostate. All angiographic and PAE procedures were performed in an interventional radiology suite equipped with a catheterization angiography laboratory (Innova 4100; GE Healthcare, Milwaukee, WI, USA) and an augmented fluoroscopy system (Vessel ASSIST; GE Healthcare), with administration of the nonionic contrast medium iodixanol (320 mg/mL, Visipaque; GE Healthcare Ireland Limited, Cork, Ireland).

All procedures were performed under local anesthesia with a unilateral femoral approach, and the prostatic arteries were catheterized with 2.0 Fr microcatheters (Progreat; Terumo, Tokyo, Japan). Each PAE was performed with 300-500 µm or with a combination of 100-300 µm and 300-500 µm of trisacryl gelatin microspheres (Embosphere Microspheres; Merit Medical Systems, South Jordan, UT, USA). Intraprocedural findings were confirmed by cone-beam computed tomography.

Patients received ciprofloxacin, 400 mg intravenously, in a single dose, before the procedure and 500 mg orally twice a day for one week thereafter. Patients were instructed to use nonsteroidal anti-inflammatory drugs, opioid analgesics, or both, as necessary, after the procedure. To lessen the effects of post-PAE syndrome, alpha blockers were maintained for 1-4 weeks after the procedure. Patients were discharged from the hospital on the same day of PAE and were followed by urologists and interventional radiologists.

### Statistical analysis

Categorical variables are expressed as absolute and relative frequencies, whereas continuous variables are expressed as means and standard deviations. The Wilcoxon signed-rank test was used in order to compare measures between baseline and 3 months of follow-up. Values of *p* < 0.05 were considered significant, and all tests were two-tailed. Statistical analysis was performed with the SPSS Statistics software package, version 23.0 (IBM Corp., Armonk, NY, USA).

## RESULTS

Between March 2013 and May 2019, 18 patients with giant prostatic hyperplasia underwent PAE. Although the mean follow-up period was 22 ± 20 months (range, 3-72 months), the outcomes were analyzed at 3 months, mainly because four patients (22.2%) were included more recently. Technical success (bilateral embolization) was achieved in 17 patients (94%). At 3 months of follow-up, one patient (5.6%) had an IPSS ≥ 8 and the indwelling catheter could not be removed in another patient (5.6%). Therefore, the clinical failure rate was 11.1%.

It is noteworthy that, at baseline, the studied population had moderate-to-severe IPSSs (range, 9-22). At 3 months after PAE, the IPSS showed an 81.3% reduction when compared with the baseline value (2.9 ± 2.8 vs. 15.73 ± 4.2; *p* = 0.001). Similar decreases were observed for the IPSS QoL item score, PSA level, and PVR. The peak urinary flow rate showed a mean increase of 11.2 mL/s, whereas prostate volume showed a mean reduction of 100.8 cm^3^ (*p* < 0.05 for both). The comparisons between the baseline values and those obtained at 3 months of follow-up are summarized in Table 2. Notably, the 11.2 mL/s mean increase in the peak urinary flow rate corresponded to a 149.73% increase over the baseline value. Also at 3 months of follow-up, one patient presented detachment of prostate tissue, accompanied by self-limited hematuria. No other specific complications were observed.

**Table 2:** Summary of changes in relevant outcome measures.

Outcome measure	Baseline Mean ± SD	3 months Mean ± SD	*P*	Change	Achange (%)
IPSS	15.7 ± 4.2	2.9 ± 2.9	0.001	-12.8	-81.3
IPSS QoL item score	5.2 ± 1.1	1.0 ± 0.7	0.005	-4.2	-80.6
PSA (ng/mL)	11.3 ± 9.0	1.82 ± 1.6	< 0.001	-9.6	-84.0
Peak urinary flow rate (mL/s)	7.5 ± 4.1	18.6 ± 8.4	0.012	+11.2	+149.7
Prostate volume (cm^3^)	252.4 ± 45.0	151.6 ± 36.0	0.001	-100.8	-40.0
PVR (mL/s)	143.7 ± 159.4	28.3 ± 14.6	0.028	-115.4	-80.3

## DISCUSSION

When seen in men, LUTS have traditionally been associated with BPH. The goal of PAE as an alternative therapeutic approach to address BPH is to relieve LUTS, as well as to slow the progression of the disease while improving QoL. Nonetheless, recent studies have demonstrated that LUTS may be caused by other pathophysiologic processes^(13)^. An increasing body of evidence suggests that inflammation is a common pathophysiological cause of LUTS and metabolic syndrome^(14,15)^. Rapid clinical improvement after PAE is common and may be due to early ischemia of the prostate gland, which reduces its volume and increases its elasticity^(16,17)^. In addition, PAE can prevent the conversion of testosterone to dihydrotestosterone (one of the factors associated with LUTS), with consequent urodynamic improvement due to resolution of the bladder outlet obstruction caused by BPH. It should be borne in mind that a prostate volume ≥ 200 cm^3^ is not common and that patients with such marked prostate enlargement may have a different natural history of disease than have those evaluated in the majority of previous studies of PAE. Therefore, this specific subgroup of patients merits further investigation.

Holmium laser enucleation of the prostate is a promising alternative for the treatment of giant prostatic hyperplasia, because it provides beneficial results and is associated with lower morbidity than is open prostatectomy. However, it has some relevant drawbacks, include its high cost, the steep learning curve, the need for specialized equipment, the long urethral instrumentation times, and the need to morcellate the laser-resected tissue that migrates into the bladder. Nevertheless, open prostatectomy involves extraperitoneal incision, necessitates blood transfusion, has a risk of neurovascular, sphincter, or rectal injury, prolongs the hospital stay, and increases catheterization time^(18)^. The guidelines established by the American Urological Association show a flowchart recommending open prostatectomy for large prostates and other surgical procedures for small and average-sized glands^(19)^. In this specific group of patients (with a prostate volume ≥ 200 cm^3^), PAE seems especially appealing because of its favorable profile in terms of complications, with a very low incidence of retrograde ejaculation and no impairment of sexual function. In addition, PAE can be performed as an outpatient procedure, allowing a faster recovery, and does not require bladder catheterization^(20)^.

In general, the results obtained in the present study are similar to those reported in previous studies of PAE, including one involving patients with a prostate volume > 80 cm^3^ and a mean baseline prostate volume of 129.4 cm^3(^^21)^. Another study described the use of PAE in treating patients with a prostate volume > 90 cm^3^, and the results were similar to those observed in our sample, the authors reporting a 32% reduction in prostate volume and an 85.2% decrease in the IPSS, also at 3 months of follow-up^(22)^. Hwang et al.^(23)^, comparing embolization particle types in a population with a mean age of 78 years, similar to that of our study population, reported a mean prostate volume of 89.4 ± 59.3 cm^3^, with a maximum of 213.1 cm^3^, and a significant (40.24%) reduction in the IPSS after the procedure (from 24.6 ± 9.7 to 14.7 ± 9.4), although the mean baseline IPSS was higher among their patients than among ours.

In one recent study^(24)^, the use of PAE was shown to be efficacious and safe in a small cohort of patients with giant prostatic hyperplasia (prostate volume ≥ 200 cm^3^), with a mean follow-up period of 5.0 ± 2.6 months. In that study, four (50%) of the eight patients evaluated had an indwelling urinary catheter and urinary retention at the time of PAE, and it was possible to remove the catheter after the procedure in three of those patients. The authors also reported that, over the course of the follow-up period, there were mean reductions of 16.7 points in the total IPSS and of 3.0 points in the IPSS QoL item score, together with a mean reduction in prostate volume of 32.5%, similar to the improvements seen in the present study. Unfortunately, the peak urinary flow rate was not reported in that study. The mean increase in the peak urinary flow rate observed in our patient sample, as it would be a useful comparative parameter to what was seen in the present investigation (11.2 mL/s; 149.73%) is superior to the 5.51 mL/s reported in a previous study of patients undergoing PAE^(20)^, being comparable to that achieved with classical surgical treatments. There is a need for further studies, involving larger cohorts, in order to confirm this particular finding.

Despite the fact that the mean prostate volume was greater in our patient sample than in those evaluated in previous studies^(25)^, there were no complications requiring specific therapy. This is in line with the complication profile described by Mathevosian et al.^(24)^, although other authors^(22)^ reported one major complication (persistent urinary tract infection requiring hospitalization) in a cohort of patients with a prostate volume > 90 g. In the present study, only one complication was observed, and that complication was a minor one-a case of prostate tissue detachment accompanied by mild LUTS and transient, self-limited hematuria. Other authors have reported cases of tissue elimination, rarely requiring specific treatment, after PAE^(26)^.

Our study has some limitations, primarily the biases inherent to the retrospective, single-center design. The short follow-up period can also be considered a limitation. Studies including larger cohorts and longer follow-up period are warranted in order to corroborate our findings.

## CONCLUSION

In patients with markedly enlarged prostates, PAE is safe and effective, providing significant clinical, imaging, and urodynamic improvements, at least in the short term.
